# Linezolid resistant coagulase negative staphylococci (LRCoNS) with novel mutations causing blood stream infections (BSI) in India

**DOI:** 10.1186/s12879-019-4368-6

**Published:** 2019-08-14

**Authors:** Gajanand Mittal, Vasundhra Bhandari, Rajni Gaind, Vandana Rani, Shimpi Chopra, Reetika Dawar, Raman Sardana, P. K. Verma

**Affiliations:** 1 Vardhman Mahavir Medical College & Safdarjung Hospital, New Delhi, 110029 India; 2 National Institute of Animal Biotechnology-DBT, Hyderabad, 500049 India; 3 Indraprastha Apollo Hospital, New Delhi, 110076 India

**Keywords:** Novel mutation, Linezolid resistance, CoNS, cfr, *S. arlettae*, India

## Abstract

**Background:**

Coagulase-negative Staphylococci (CoNS) have emerged as a major causative agent of blood-stream infections (BSI). Linezolid (LZD) is currently used for treating glycopeptide and methicillin-resistant staphylococci. It is important to understand the resistance mechanism and probable transmission of LZD resistant (LR) CoNS within the hospital.

**Methods:**

Clinically significant LRCoNS from patients with BSI were characterized using MALDI-TOF and 16S *rRNA* gene sequence analysis. Antimicrobial susceptibility and MIC of vancomycin and LZD were determined. LZD resistance mechanisms using PCR for the *cfr* gene and mutation in the V domain of the 23S *rRNA* gene were studied.

**Results:**

The MIC of LZD ranged from 8 to 32 μg/ml. LR was observed in three different CoNS species from diverse locations within the hospital. The *cfr* gene was identified in all the isolates. Sequence analysis of V domain region of 23S rRNA gene confirmed mutation in single copy among 12/15 isolates with novel mutations: G2614 T and C2384T. All infections were nosocomially acquired and LZD resistance was emerging in the absence of prior LZD use. Horizontal spread of resistant isolates and *cfr gen*e among diverse species were the probable mechanisms of transmission.

**Conclusion:**

The study highlights the novel mutations associated with LRCoNS and the importance of surveillance & transmission pathway within the hospital. It also systematically discusses the published information on LRCoNS.

## Background

Coagulase-negative Staphylococci (CoNS) are normal commensals of the skin and mucous membranes and have emerged as the important cause of hospital-acquired infections [1, 2]. They are the most common cause of healthcare-associated blood stream infection (BSI) for many years, partly because of an increase in the number of hospitalized immuno-compromised patients, the increased use of indwelling medical devices, such as central venous catheters and other prosthetic implants [1]. The clinical significance of species other than *S. epidermidis* has been increasingly recognized in recent years [2]. As the pathogenic significance increases, it becomes important to learn about the epidemiology and pathogenic potential of individual species [2]. Species identification is also important as certain species like *S. epidermidis* and *S. haemolyticus* are resistant to multiple antibiotics [2]. Routine species identification may thus better define the clinical spectrum of disease caused by CoNS. Currently, there is a paucity of data on the clinically significant CoNS species as conventional identification methods are labor-intensive [3]. Methicillin-resistant CoNS are cross-resistant to all other β-lactam antibiotics and CoNS with decreased susceptibility to glycopeptides have been reported [1]. Management of CoNS infections is thus challenging because of the associated risk factors and the multiple drug resistance, which narrows therapeutic options [4].

Linezolid (LZD) is a synthetic drug of oxazolidinone class of antibiotics, which is approved for treatment of severe bacterial infections in adults caused by drug-resistant gram-positive bacteria, such as multidrug-resistant *S. aureus,* coagulase negative staphylococci, penicillin-resistant *Streptococcus pneumoniae* and vancomycin-resistant Enterococci (VRE) [5–7]. When introduced, it was claimed that LZD has no cross-tolerance against other antibiotics and resistance developed rarely, due to its unique mechanism of action [8, 9] However, a year after the introduction, the first LZD resistant clinical strain appeared in 2001 [5]. Despite a decade of its clinical use, resistance to LZD has remained stable and extremely low with only sporadic cases being reported mostly from the USA and Europe [10]. Resistance may arise during therapy, especially in deep-seated infections treated over prolonged courses [6]. The bacteriostatic action of antibiotic blocks protein synthesis by interfering with the positioning of A-site *tRNA* in the peptidyl transferase centre of 23S *rRNA* in the 50S ribosomal subunit [5].

Resistance to LZD is primarily caused by mutations in the domain V of 23S *rRNA* gene or the gene *cfr* (chloramphenicol florfenicol resistance) [5]. Co-occurrence of *cfr*-mediated resistance and mutational resistance has also been documented and pose a therapeutic concern [5]. The G2576 T mutation in the *23S rRNA* is most common, other mutations including T2500A, G2603 T, C2534T, T2504A, G2447 T, G2215A, and G2631 T, have been reported among clinical staphylococcal isolates [7, 11, 12]. Resistance mediated by *cfr* gene is also of great concern as it is usually plasmid-borne and can be easily disseminated to susceptible population [9]. The *cfr* gene also encodes resistance to a group of chemically distinct antibiotics such as phenicols, lincosamides, pleuromutilins and streptogramin-A leading to a multidrug-resistant phenotype [5, 6]. The *cfr* gene was first seen in veterinary isolates of *Staphylococcus warneri, Staphylococcus sciuri, Staphylococcus hyicus,* and *S. aureus* perhaps associated with the veterinary use of phenicols [6]. Till date, the linezolid resistance among CoNS (LRCoNS) has been reported from various countries including North America (USA, Mexico), South America (Brazil), Europe (Greece, Spain, Italy, France, and Ireland), and Asia [10]. There are limited reports of characterization of the mechanism of resistance from India [5].

In the current study, species distribution, susceptibility profile and the mechanism of LZD resistance among LRCoNS were studied. An attempt was made to study the clinical profile of patients with BSIs caused by LRCoNS and study the probable transmission pathway within the hospital.

## Methods

### Patient information, bacterial isolation, and species characterization

Clinically significant LRCoNS (LZD MIC ≥8 μg/ml) isolated from patients with BSI admitted at Safdarjung Hospital, New Delhi, India from August 2013 to August 2015 were studied. Only one isolate per patient was included in the study. CoNS were identified by Gram stain, the presence of catalase, and negative tube coagulase test [5]. The isolates were characterized by Matrix Assisted Laser Desorption/Ionization Time-of-Flight (MALDI-TOF Vitek MS, bioMerieux, France). Demographic data, clinical history including prior antibiotic therapy, invasive procedures, and co-morbid conditions were recorded through chart review. Isolates were classified as nosocomial if the sample was collected more than 48 h following admission to the hospital [6]. Standardized criteria for the diagnosis of nosocomial infections were used to determine the clinical significance of test isolates. Bacteremic episodes were classified as true infection if: multiple positive blood cultures, a positive blood culture along with a positive culture with the same organism from another site, and bacteremia associated with systemic symptoms (fever [temperature > 38.5 °C], hypotension [systolic blood pressure < 90 mm/Hg], and leukocytosis [> 13,000 cells/mL]) not attributed to other causes [6].

To study the transmission pathway line listing of the patients with LRCoNS BSI was done using the date of admission, date of positive blood culture, date of discharge or outcome and is shown in Table 1 and Fig. 2.

**Table 1:** The clinical profile, details of hospitalization, outcome of patients and molecular characterization of LRCoNS

S. NO.	^a^AGE GROUP/SEX	WARD	HISTORY/DIAGNOSIS	DEVICE	SPECIES	MIC (μg/ml)		Resistance pattern	Mechanism of Resistance	
						LZ	VA		*cfr* gene	23S rRNA Mutation
1^b^	1–5 /F	Paediatric	Congenital Cystic Adenomatoid Malformation & Respiratory Failure	ICD, PC	*S. arlettae*	8	2	CN, E, CD, G, CIP, COT	POS	G2614 T
2	< 1/F	Paediatric	VSD, Hepatomegaly, Pneumonia	PC	*S. arlettae*	16	2	CN, E, CD, CIP, COT	POS	G2614 T C2384T
3	21–40/M	ICU	Hepatic abscess	V, PC	*S.haemolyticus*	8	2	CN, E, CD, G, CIP, CH, COT	POS	G2614 T
4^b^	21–40/M	ICU	Perforation Peritonitis With Septic Shock	V, PC	*S.haemolyticus*	32	4	CN, E, CD, G, CIP, CH, COT	POS	G2614 T
5^b^	41–60/M	Medicine	Diabetes MellitusChronic alcoholic with hepatitis	PC	*S.cohnii*	32	2	CN, E, CD, G, CIP, CH,COT	POS	NIL
6	1–5 /F	Paediatric	Pneumonia	PC	*S.haemolyticus*	32	4	CN, E, CD, G, CIP, CH,COT, TC	POS	G2614 T
7	21–40/M	ICU	fracture OF Inferior pelvic remi, acetabulam,ileum, hemo-peritoneum, Pleural effusion	V, PC	*S.haemolyticus*	32	4	CN,E,CD, G, CIP, CH,COT, TC	POS	G2614 T
8	21–40/F	ICU	Pulmonary edema With Amniotic Fluid Embolism With ARF	V, PC	*S.cohnii*	8	1	CN,E,CD, G, CIP, CH, COT, TC	POS	C2384T
9	21–40/F	Respiratory medicine	Pneumonia	PC	*S.haemolyticus*	32	2	CN,E,CD, G, CIP, CH, COT, TC	POS	G2614 T
10	61–80/F	ICU	Closed traumatic fracture neck femur, DVT, Pulmonary Embolism	V, PC	*S.haemolyticus*	16	2	CN,E,CD, G, CIP, CH, COT, TC	POS	G2614 T
11	11–20/M	ICU	Opium Poisoning	V, PC	*S.haemolyticus*	32	2	CN,E,CD, G, CIP, CH, COT, TC	POS	G2614 T
12	41–60/F	Oncology	Carcinoma Ovary With Metastasis	PC	*S.haemolyticus*	32	2	CN,E,CD, G, CIP, CH, COT, TC	POS	G2614 T
13	21–40/F	Obstetrics	Preterm Baby With PROM	PC	*S.haemolyticus*	8	1	CN, E, CD, G, COT, TC	POS	G2614 T
14	41–60/F	ICU	Head Injury With GCS Score 2	V, PC	*S.arlettae*	8	2	CN,E,CD, G, CIP, CH, COT, TC	POS	NIL
15	11–20/F	ICU	Snake bite	V, PC	*S.cohnii*	8	2	CN,E,CD, G, CIP, CH, COT, TC	POS	NIL

^a^ Age group in Years < 1, < 5, 5–10, 11–20,21-40,41-60,61–80,> 80

^b^ patient expired, *M* Male, *F* Female, *VSD* Ventricular septal defect, *ARF* Acute renal failure, *DVT* Deep vein thrombosis, *PROM* Premature rupture of membrane, *GCS* Glasgow coma score, *V* Ventilator, *PC* Peripheral catheter, *ICD* Intercostal drain, *POS* Positive, *LZ* Linezolid, *VA* Vancomycin, *CN* Cefoxitin, *E* Erytromycin, *CD* Clindamycin, *G* Gentamicin, *CIP* Ciprofloxacin, *CH* Chloramphenicol, *COT* Trimethoprim-sulfamethoxazole, *TC* Teicoplanin

GenBank Accession numbers of the 23 s rRNA gene sequence of the isolates in which mutation was observed are KY952716, KY952715, KY952717, KY952718, KY952719, KY952720, KY952714, KY952721, KY952722, KY952723, KY952724 and KY952725 in order of the serial number in the table

### Antibiotic susceptibility assay

Antimicrobial susceptibility testing was done using disc diffusion assay (cefoxitin 30 μg, erythromycin 15 μg, clindamycin 2 μg, gentamicin 10 μg, ciprofloxacin 5 μg, chloramphenicol 30 μg, trimethoprim-sulfamethoxazole 1.25/23.75 μg, and teicoplanin 30 μg). All discs were procured from Oxoid Ltd., Basingstoke, UK. Minimum inhibition concentrations (MIC) of LZD (Sigma-Aldrich, USA) and vancomycin (Sigma-Aldrich, USA) were determined by resazurin dye based microbroth dilution. All assays were performed as per the guidelines of CLSI, 2015 [13, 14]. *S. aureus* ATCC 29213 and *S. aureus* ATCC 700699 (Mu50) were used as a control strains for all susceptibility assays.

### DNA extraction

DNA was isolated using Wizard genomic kit (Promega, Madison, WI, USA) from 2 ml of overnight grown bacterial cultures in Mueller Hinton broth. Cells were pelleted at 10,000X *g* for 3 min and washed with 1X PBS twice at 8000X *g*, 3 min and suspended in 500 μl of 50 mM EDTA, lysostaphin (100 μg/ml) and lysozyme (100 μg/ml) for 1 h at 37 °C followed by manufacturer’s instructions.

### *cfr* gene amplification using polymerase chain reaction

*cfr* gene was amplified from the extracted DNA using forward 5′-TGA AGT ATA AAG CAG GTT GGAG-3′ and reverse 5′-ACC ATA TA A TTG ACC ACA AGC AG-‘3 primer set as described previously [15].

### PCR amplification and sequence analysis of 23S rRNA gene copies and domain *V* region

The domain *V* region was amplified using forward 5′-GCGGTCGCCTCCTAAAAG-3′ and reverse 5′-ATCCCGGTCCTCTCGTACTA-3′ primers. Amplification started with an initial denaturation at 95 °C for 5 min followed by 30 cycles of denaturing (94 °C, 30s), annealing (55 °C, 30s), and extending cycles (72 °C, 30s); and a final extension of 10 min at 72 °C. PCR products corresponding to 420 base pairs were confirmed by running on 2% agarose gel. The amplified product was eluted and purified using gel purification kit and sequenced. Five copies of the *23S rRNA* gene were amplified using specific primers for each gene copy as described earlier [16]. PCR products were analyzed using agarose gel electrophoresis. Sequence analysis was done by Sanger sequencing using DNA Star software at Bioserve Pvt. Ltd. Hyderabad, to check for mutations. Sequences of novel mutations were uploaded to GenBank.

### Pulse field gel electrophoresis (PFGE)

PFGE was performed for nine *S. haemolyticus* isolates as described earlier [17]. Briefly, the Genomic DNA was digested with SmaI and the DNA fragments were separated in a 1% agarose gel using BioRAD,CHEF Mapper System III (BioRad). *Staphylococcus aureus* ATCC25923 was used as a reference control. PFGE patterns were further analysed using a temporary BioNumerics evaluation license from Applied Maths. Permission to publish the PFGE data was obtained from Applied Maths.

## Results

### Clinical profile of patients with LRCoNS

LZD resistance was observed among 15 clinically significant CoNS isolates from patients with clinical sepsis. The clinical profile and outcome of patients with LRCoNS BSI are shown in Table 1. Mean duration of hospital stay prior to sepsis was 24 days (3–90 days). The chart review and patient history suggested that all the 15 LRCoNS were nosocomially acquired in patients admitted to pediatric (*n* = 3), medicine (*n* = 1), oncology (n = 1), obstetrics and gynaecology (n = 1), respiratory medicine (n = 1) ward and ICU (*n* = 8) (Table 1). LRCoNS infections were observed in five males with a mean age of 31.5 years (range 18–42 year), three female children with a mean age of 1.3 years (range 8 month – 2 year) and seven females with a mean age of 38.4 year (range 12 year –80 year). Overall, mean age was 30 years (range 8 month–80 year). All patients had co-morbid conditions (Table 1) and were on the peripheral catheter. In addition, all patients admitted to ICU were on a ventilator and one pediatric patient was on intercostal drainage. None of these patients had received LZD therapy or other agents that might select *cfr,* such as clindamycin or chloramphenicol before sample collection. Mortality was observed among 20% (3/15) of the patients.

### Species identification and PFGE analysis

**Fig. 1:**
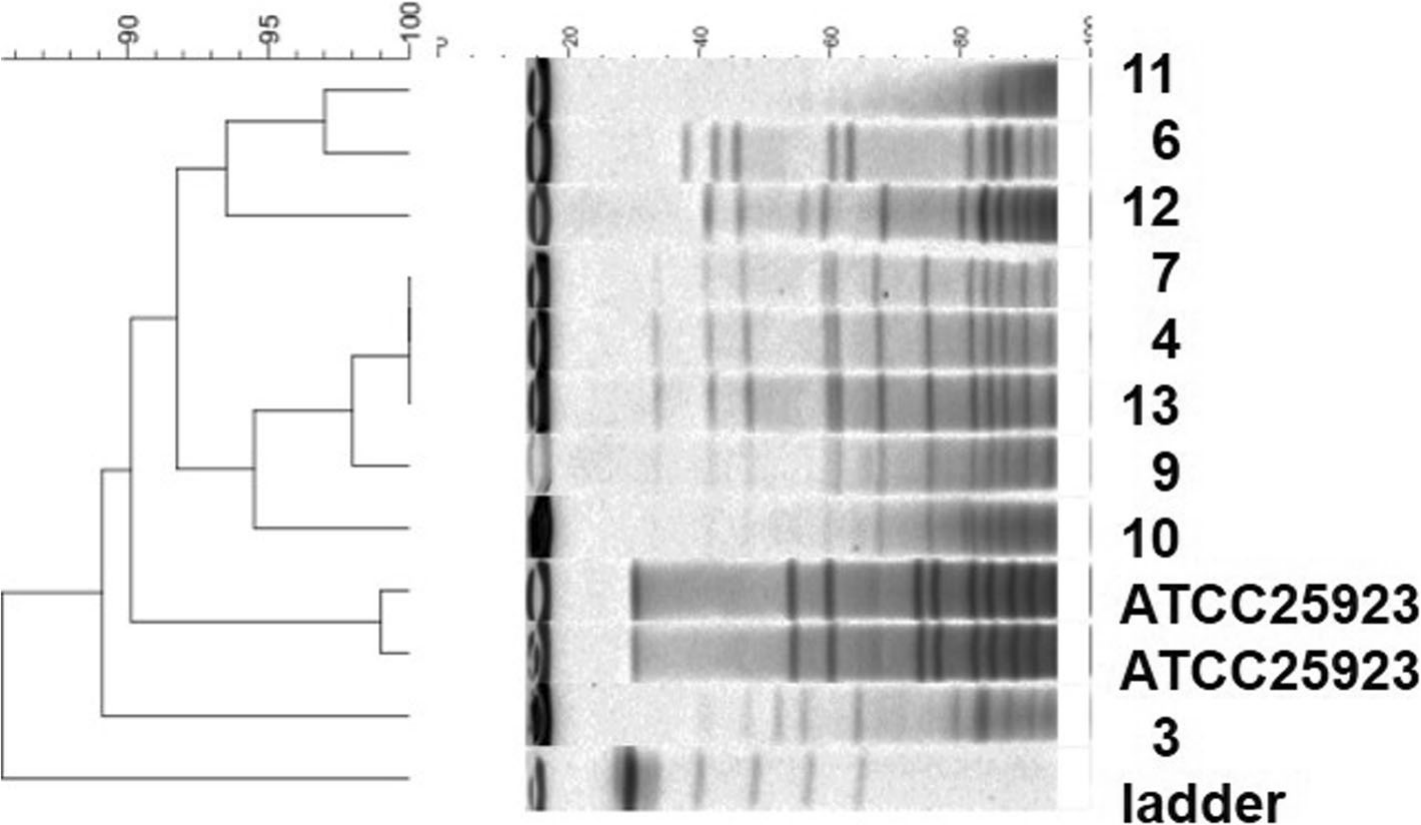
Dendrogram using PFGE profiles of *SmaI*-digested genomic DNA of nine S. *haemolyticus* clinical isolates. ATCC 25923 was used as reference control and the numbers (3, 4, 6, 7, 9, 10, 11, 12, 13) denotes the patient number from which *S. haemolyticus* was obtained as described in the manuscript

LZD resistance was observed among three different species; *S. haemolyticus* (*n* = 9), *S. cohnii* (*n* = 3) and *S. arlettae* (n = 3). The PFGE analysis of the *S. haemolyticus* isolates revealed that 6 out of 9 isolates showed a distinguishable pattern from each other (Fig. 1). However,2 isolates from ICU patients (4,7) and one from Obstretics Departmnet (13) showed similar patterns (Table 1 and Fig. 1).

### Antibiotic susceptibility

All LRCoNS (LZD MIC range 8-32 μg/ml) were also resistant to cefoxitin, erythromycin, clindamycin, trimethoprim/sulfamethoxazole as determined by disc diffusion method. Resistance to chloramphenicol, gentamicin, and ciprofloxacin was observed in 80% (12/15), 90.3% (14/15) and 90.3% (14/15) isolates respectively. However, all isolates were susceptible to vancomycin (MIC range 1-4 μg/ml) and only 33% of the strains were susceptible to teicoplanin.

### Linezolid resistance determinants and correlation with MIC

All strains were positive for *cfr* gene with a specific amplification of 746 base pair. Mutations were detected in 12/15 isolates in a single copy of *23S rRNA* gene (Table 1). Novel mutations, G2614 T and C2384T were observed. A single G2614 T mutation was detected in 10/15 isolates and included *S. haemolyticus* (*n* = 9; LZD MIC range 8-32 μg/ml) and *S. arlettae* (*n* = 1; LZD MIC 8 μg/ml). C2384T mutation was observed in one isolate of *S. cohnii* (LZD MIC 8 μg/ml) and two mutations were observed (G2614 T & C2384T) in one isolate of *S. arlettae* (LZD MIC 16 μg/ml). Among 3 isolates (2 *S. cohnii*, 1 *S. arlettae*) no mutation was identified (LZD MIC 8-32 μg/ml).

## Discussion

Infections caused by CoNS are endogenous and skin and skin and mucous membranes colonization are the key source of infections [18]. These organisms have relatively low virulence but are increasingly recognized as agents of clinically significant BSI and other sites because of their tendency to form biofilms on medical devices that penetrate skin surfaces [19]. Various studies have documented that drastic changes in patient populations (increased numbers of premature newborns and elderly), multi-morbid, chronically ill, and, often, immune-compromised patients and as well as the increasing use of invasive devices have predisposed to infections caused by CoNS [18]. Therapeutically, CoNS are challenging due to the large proportion of methicillin-resistant strains and increasing numbers of isolates with less susceptibility to glycopeptide. Most studies on CoNS do not distinguish among different species, therefore the factual impact of infrequently occurring species might be under-reported [18]. Further species identification is important to study the source of infection, monitor outbreaks and their role in clinically significant infections [20].

In the current study, CoNS sepsis was observed predominantly in ICU patients and all patients had co-morbid conditions with invasive procedures. LZD resistance was observed in 3 different CoNS species; most common being *S. haemolyticus*, followed by *S. cohnii* and *S. arlettae.* This is the first report of LZD resistance in *S. arlettae* mediated by both *cfr* gene and novel mutations in domain *V* region of the *23S rRNA* gene*.* The most commonly observed mutation, G2576 T in domain *V* region of the *23S rRNA gene*, was not detected in our study. However, novel mutations were observed and included G2614 T in *S. haemolyticus* and *S. arlettae* and C2384T in *S. cohnii* and *S. arlettae*.

The LZD MIC among LRCoNS ranged from 8 to 32 μg/ml and there was no correlation between MIC, mechanism/mechanisms of resistance, type of mutation observed (Table 1). This is in contrast to the higher MIC reported in another study (MIC > 256 μg/ml) [21]. Staphylococci have multiple copies of the gene that encodes domain *V* region of the *23S rRNA* gene, the location of the target for LZD [22]. A gene dosage effect has been described, whereby LZD MICs increase with the number of gene copies that have mutations [22]. In the present study, mutations were observed in only one copy of the *23S rRNA* gene, which may explain the low MIC. However, novel mutations (G2614 T, C2384T) were detected and their effect on LZD MIC was difficult to determine because of the presence of the *cfr* gene, which was detected in all isolates. The heterogeneity of MIC even among isolates with the same number of mutant alleles suggests that other mechanism of LZD resistance might be affecting linezolid susceptibility.

The LRCoNS were isolated from diverse locations within the hospital (Table 1). Among three *S. arlettae* isolates, two were isolated from patients admitted to pediatric wards in the month of May 2013 and July 2013 (Table 1). Although both harbored *cfr* gene but were distinct with different antibiotic profile and mutations in domain *V* region of the *23S rRNA* gene. The third isolate was isolated from an adult patient admitted to ICU in May 2015 harboring only *cfr* gene. As *S. arlettae* were isolated from different locations with distinct phenotypes, these facts suggest that different clones of *S. arlettae* were circulating in the hospital. Among the three *S. cohnii* isolates, two were from ICU in March 2015 and June 2015 with an identical antibiotic profile, however, the latter isolate had no mutation in the domain *V* of the *23S rRNA gene*. The third isolate was isolated from a patient in medicine department in July 2015 also showed no mutation. Among the 9 isolates of *S. haemolyticus*, all demonstrated the same novel mutation (G2614 T), 5/9 isolates were from ICU and other 4/9 isolates were from diverse locations in the hospital. Seven different PFGE banding pattern were observed among the nine *S. haemolyticus* isolates obtained from various departments. Only three isolates obtained from patients (4, 7 & 13) showed a similar pattern indicating emergence from the same clone. These findings suggest that different clones of LRCoNS may be circulating in the hospital which is in contrast to other published studies where all isolates showed similar PFGE banding pattern [17, 23, 24].

**Fig. 2:**
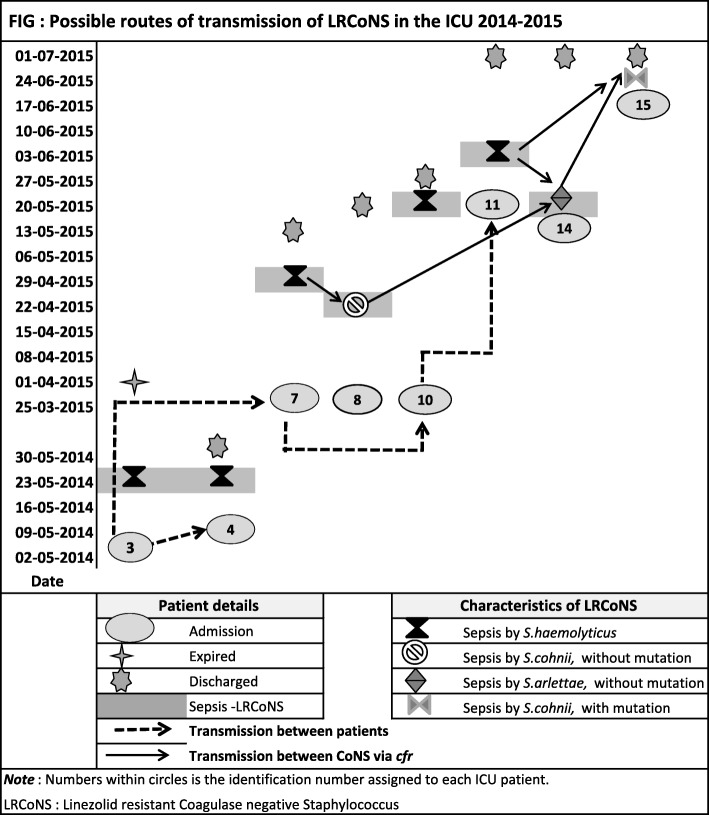
Possible routes of transmission of LRCoNS in the ICU 2014-2015

The majority (8/15) of LRCoNS was isolated from the ICU and belonged to three diverse species. Probable transmission routes in the ICU are explained diagrammatically in Fig. 2. All infections were nosocomially acquired as sepsis developed after 48 h of admission. Patient 3, 4,7,10 and 11 (Fig. 2) developed sepsis due to *S. haemolyticus*. Antibiograms of isolates patient 3 and 4 differed from patient 7,10 and 11, however all isolates have a G2614 T mutation and *cfr* gene. The PFGE suggested all isolates except those from patient 3 and 4 were indistinguishable. Moreover, the time line of events from admission to discharge/death suggests probable spread through cross transmission within the ICU due to a breach of infection control practices. The prolonged ICU stay, associated comorbid conditions and use of invasive devices may have further contributed to cross transmission. Patient 8, 14 and 15 (Fig. 2) developed BSI due to LR *S. cohnii* (*cfr* gene with mutation), *S. arlettae* (*cfr* gene without mutation) and *S. cohnii* (*cfr* gene without mutation) which were phenotypically distinct and with different LZD resistance mechanism. The possibility of horizontal transmission among different CoNS species mediated by *cfr* gene from LR *S. haemolyticus* cannot be ruled out. These findings suggest that linezolid resistance emerged in ICU both due to cross-transmission between patients and horizontal transfer of LZD resistance mediated by *cfr* gene among different CoNS species. However, limitation of this study is that due to lack of resources and involvement of diverse species of CONS, we could not confirm the transmission pathway using suitable typing method for species other than LR *S. haemolyticus*.

In earlier reports, previous administration of LZD has been reported to be an independent predictor of LZD resistance in CoNS [21], interestingly, in our study patients did not receive LZD therapy prior to being infected with the resistant strain. Patients having LRCoNS infections without prior exposure to the linezolid have been reported earlier [21, 25]. The source of the LRCoNS remained undetermined, however, the clonal spread has been reported to occur within the hospitals [25]. In the present study, the possibility of nosocomial transmission from patients colonized with LRCoNS as part of the skin flora following LZD exposure (both in a hospital or outside prior to admission) needs to be investigated which could have been the source of infections. An alternative explanation is that selection for de novo resistance in a prevalent nosocomial clone of linezolid-susceptible CoNS has occurred.

LRCoNS were reported worldwide, including North America, South America, European and Asia. The mechanisms for LZD resistance were L3/L4 mutation, mutations in the 23S rRNA and the presence of a transmissible *cfr* ribosomal methyltransferase. In all reported cases, strains including *S. haemolyticus, S. cohnii*, *S. epidermidis*, *S. hominis, S. capitis, S. sciuri, S. lugdunensis, S. simulans* and *S. kloosii* were isolated from an aseptic sample, which included blood, pus, CSF and catheter tips. In our study, *S. haemolyticus* was the predominant species and novel mutations were reported. There have been limited reports of *S. arlettae.* Dinakaran et al., reported a case of *S. arlettae* isolated from the blood of a cardiovascular disease patient [26]. Our study suggests that *S. arlettae* could be an emerging pathogen.

Interestingly, all the isolates reported were susceptible to vancomycin (MIC range 1-4 μg/ml) and 66% isolates were resistant to teicoplanin. *S. haemolyticus* has emerged as a nosocomial pathogen on account of its ability to attain high-level resistance to many antibiotics including glycopeptides. We observed 77% of *S. haemolyticus* resistant to teicoplanin in absence of prior exposure to this antibiotic. This linezolid-teicoplanin-resistant *S. haemolyticus* (LTR-SH) is a threat. There is thus an urgent need to identify CoNS to species level along with antibiotic profile before initiating therapy, as these are rarely determined in hospital settings.

Surveillance of LZD resistance in *Staphylococcus* will not only help in proper antibiotic usage but will also avoid emergence of multidrug resistant bacteria [27]. Mutational resistance to linezolid is troublesome in clinical practice, but the acquisition of the *cfr* gene is a more worrying threat because of its rapid spread and horizontal transmission between species [28]. This gene, originally found in animal strains, is now increasingly reported in humans and therefore attention should be paid to the fact that these strains might also be selected under treatment with phenicols or macrolides; this could be due to co-selection and might multiply the risk of development of linezolid-resistant strains [29]. Though the LZD-resistant *Staphylococcus* is still sporadic now, the prolonged hospital stays; frequent interventions and misuse of antibiotics may accelerate the dissemination of LZD resistance *Staphylococcus* [27]. So, we suggest that an effective nosocomial infection control strategy, which includes reinforcement of hand hygiene, judicious use of antibiotics and screening of patients colonized with LR-CoNS, should be established to prevent the further spread of multi-drug resistant LR-CoNS strains and to preserve the therapeutic efficacy of this important antimicrobial [28].

## Data Availability

The data generated during the current study are available on GenBank nucleotide sequence database (https://www.ncbi.nlm.nih.gov/).

## Abbreviations

BSI: Blood stream infection
CoNS: Coagulase negative Staphylococci
LRCoNS: Linezolid Resistant Coagulase Negative Staphylococci
LZD: Linezolid
